# Novel Mechanisms of ALK Activation Revealed by Analysis of the Y1278S Neuroblastoma Mutation

**DOI:** 10.3390/cancers9110149

**Published:** 2017-10-30

**Authors:** Jikui Guan, Yasuo Yamazaki, Damini Chand, Jesper R. van Dijk, Kristina Ruuth, Ruth H. Palmer, Bengt Hallberg

**Affiliations:** 1 Institute of Biomedicine, Department of Medical Biochemistry and Cell Biology, Sahlgrenska Academy, University of Gothenburg, SE-40530 Göteborg, Sweden; Jikui.Guan@gu.se (J.G.); yasuo.yamazaki@ncvc.go.jp (Y.Y.); damini.chand@einstein.yu.edu (D.C.); Jespervd90@gmail.com (J.R.v.D.); Ruth.Palmer@gu.se (R.H.P.); 2 Department of Molecular Biology, Umeå universtiy, SE-901-87 Umeå, Sweden; Kristina.Ruuth@molbiol.umu.se

**Keywords:** ALK, kinase activation, activation loop, gain-of-function, mutation analyses, Insulin receptor, NPM-ALK, oncogene, neuroblastoma

## Abstract

Numerous mutations have been observed in the Anaplastic Lymphoma Kinase (ALK) receptor tyrosine kinase (RTK) in both germline and sporadic neuroblastoma. Here, we have investigated the Y1278S mutation, observed in four patient cases, and its potential importance in the activation of the full length ALK receptor. Y1278S is located in the 1278-YRASYY-1283 motif of the ALK activation loop, which has previously been reported to be important in the activation of the ALK kinase domain. In this study, we have characterized activation loop mutations within the context of the full length ALK employing cell culture and *Drosophila melanogaster* model systems. Our results show that the Y1278S mutant observed in patients with neuroblastoma harbors gain-of-function activity. Secondly, we show that the suggested interaction between Y1278 and other amino acids might be of less importance in the activation process of the ALK kinase than previously proposed. Thirdly, of the three individual tyrosines in the 1278-YRASYY-1283 activation loop, we find that Y1283 is the critical tyrosine in the activation process. Taken together, our observations employing different model systems reveal new mechanistic insights on how the full length ALK receptor is activated and highlight differences with earlier described activation mechanisms observed in the NPM-ALK fusion protein, supporting a mechanism of activation more in line with those observed for the Insulin Receptor (InR).

## 1. Introduction

Anaplastic lymphoma kinase was first discovered in 1994 as a fusion partner for nucleophosmin (NPM) in anaplastic large cell lymphoma (ALCL) [1,2]. Activation of the Anaplastic Lymphoma Kinase (ALK) fusion proteins is driven by oligomerisation via the fusion partner, which results in autophosphorylation of the kinase domain and subsequent downstream signaling [3]. ALK has since been associated with a plethora of human malignancies, including both familial and sporadic neuroblastoma, which is a childhood cancer of the sympathetic nervous system [3,4,5,6]. Neuroblastoma is a heterogenous disease with a variety of chromosomal rearrangements, such as amplification of the *MYCN* gene (24% of all cases), deletion of parts of chromosomes 1p and 11q, gain of parts of 17q, and triploidy [7,8,9]. Characterization of the different point mutations in ALK observed in neuroblastoma patients has led to segregation of mutations into three classes; ligand independent, ligand dependent and kinase dead forms of receptor [5,10]. The majority of these ALK point mutations are localised in the kinase domain of ALK, and include the three hot-spot mutations at residues F1174, F1245, and R1275 [3,7]. The mechanisms underlying activation of the full length ALK RTK remain enigmatic; however, recent identification of the ALKAL ligands [11,12] together with structural studies of the kinase domain have increased our understanding [13,14].

One of the earliest reports concerning the substrate specificity of ALK examined the importance of the triple tyrosine motif (1278-YXXXYY-1283) in the activation loop, a feature similar to other members of the Insulin receptor (InR) family [15]. The ALK activation loop contains a 1278-YRASYY-1283 motif that can be compared with 1158-YETDYY-1163 in the InR activation loop. The importance of the ‘RAS’ (Arg-Ala-Ser), as opposed to the ‘ETD’ (Glu-Thr-Asp) of the InR motif has been reported in studies of baculovirus produced ALK kinase domain, where the residues between the tyrosines have been shown to contribute to ALK activation loop auto-phosphorylation efficiency [16]. The authors also reported a preference for the initial tyrosine in the motif—Y1278—as the first tyrosine in the NPM-ALK fusion protein to undergo autocatalytic phosphorylation [16]. This, is in contrast to that reported for the InR, where the second tyrosine (Y-1162) is phosphorylated followed by the third (Y-1163) before finally the first tyrosine (Y1158) in the activation loop to undergo autocatalytic phosphorylation [15,17]. A subsequent study examined and confirmed the importance of the first tyrosine in the activation loop 1278-YRASYY-1283 motif in the context of the NPM-ALK fusion protein [18]. This report also indicated that Y1278 is important for the transformation activity of NPM-ALK and interaction of ALK with STAT3 [18]. Mutation of Y1278 has been reported in four neuroblastoma cases (COSMIC) [19,20,21]. In these patients, tyrosine 1278 is mutated to a serine residue—Y1278S—in the context of the full length ALK receptor and displays constitutive kinase activity. More insight into the role of Y1278 was proposed with the solving of the kinase domain structure of ALK [13,14]. This structural work highlighted a tight interaction in the inactive form of ALK between unphosphorylated tyrosine at position 1278, in the 1278-YRASYY-1283 motif of the activation loop, and a cysteine at position 1097, in the β-turn [13,14]. These reports suggested that either the mutation of Y1278 to serine or phosphorylation of Y1278 upon activation would result in the loss of stabilizing hydrogen bond with C1097, leading to a subsequent shift in the αC-helix thereby facilitating the activation of kinase domain of ALK. 

Here, we investigate the three tyrosine residues of the activation loop and the suggested interaction between Y1278 and C1097 in cell culture and *Drosophila* model systems. We show here that, in contrast to results reported for the activation of the NPM-ALK fusion protein, phosphorylation of Y1283 in full length ALK appears to be necessary for the activation of full length ALK kinase. The Y1278S neuroblastoma mutation is sufficent to activate the ALK kinase domain, however the previously proposed regulatory Y1278:C1097 hydrogen bond is not important to maintain ligand-dependent activation. Based on these results, we propose that the activation loop of the full length ALK receptor is mechanistically more similar to that of the InR than the NPM-ALK fusion protein.

## 2. Results

### 2.1. The Y1278S Neuroblastoma ALK Mutation Results in Ligand Independent Activation

**Figure 1:**
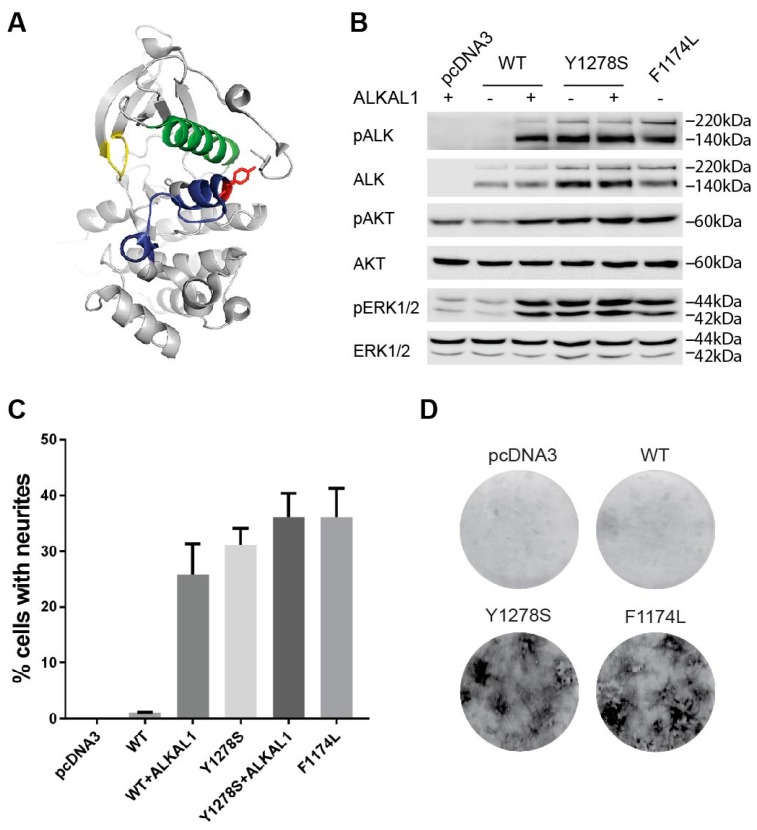
Characterization of ALK-Y1278S. (**A**) Ribbon diagram of the inactive Anaplastic Lymphoma Kinase (ALK) kinase domain indicating the location of Y1278 (red stick) in the activation loop (dark blue). αC helix: green; glycine rich P-loop: yellow. Image was generated with PyMOL (DeLano Scientific) using PDB:3LCT; (**B**) The Y1278S mutation mediates a ligand-independent activation of ALK as shown by immunoblotting of pALK (Y1604) and downstream pAKT and pERK1/2. Total ALK, AKT, and ERK1/2 were used as internal controls. Blots were representative of three independent experiments; (**C**) Neurite outgrowth of PC12 cells as a readout for ALK activity was performed with wild type ALK and the indicated ALK mutants in the absence or presence of the ALKAL1 ALK ligand. PC12 cells transfected with vector alone were employed as negative control. PC12 cells expressing activating ALK-F1174L were used as positive control. Bars represent mean percentage ± STD of neurite-carrying cells among GFP-positive cells from three independent experiments; (**D**) Representative focus formation assays for NIH3T3 cells transfected with pcDNA3 empty vector, wild type ALK, ALK-Y1278S, or ALK-F1174L.

Mutation of tyrosine 1278 to serine—Y1278S—in the activation loop of the ALK has been reported in four independent neuroblastoma cases (http://cancer-beta.sanger.ac.uk/cosmic/mutation/overview?id=28058) (Figure 1A). In order to initially characterize the nature of the ALK-Y1278S mutation, we investigated its activity in cell culture systems. Transient transfection of ALK-Y1278S in PC12 cells led to ligand independent phosphorylation/activation of ALK itself and the downstream targets AKT and ERK1/2, to levels similar to those of the previously characterized gain-of-function ALK-F1174L mutation (Figure 1B). In addition to activation of ERK1/2 we also observed robust induction of neurite outgrowth in PC12 cells, which are comparable to that seen on expression of ALK-F1174L (Figure 1C) [11]. PC12s are a clonal rat adrenal pheochromocytoma cell line with enteric cell origin, which has the ability to differentiate and extend neurites upon extended ERK1/2 stimulation [22]. We and others have previously shown that activation of both human and mouse ALK triggers differentiation of PC12 cells into sympathetic-like neurons, a process that is characterized by extension of neurites [10,23]. This assay offers a convenient read out for ALK activity in vitro. As controls we employed transient transfected wild type ALK, which upon stimulation with the ALKAL1 ligand mediates neurite outgrowth and ALK-F1174L (Figure 1C). The ALK-Y1278S mutation was also further activated upon stimulation with the ALKAL1 ligand, as suggested previously [11] (Figure 1C). Furthermore, ALK-Y1278S expression resulted in focus formation in an NIH3T3 transformation assay, in contrast to either the wild type ALK or empty vector controls (Figure 1D). Thus, the ALK-Y1278S mutant behaves as a ligand-independent receptor, inducing activation of downstream targets and neurite outgrowth.

### 2.2. Sensitivity of ALK-Y1278S to ALK Inhibitors

**Figure 2:**
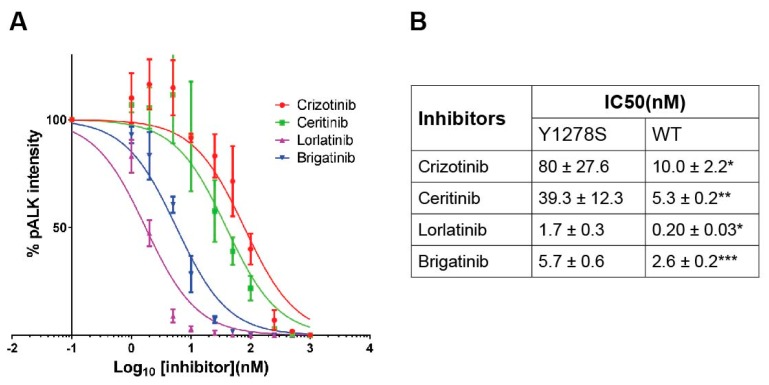
Inhibition profiling of ALK-Y1278S with ALK inhibitors. (**A**) PC12 cells expressing ALK-Y1278S were treated with serial dilutions of ALK inhibitors as indicated. Phosphorylation of ALK was detected with anti-pALK (Y1604) antibody and total ALK was used as internal control. Data were normalized to non-treated controls as percentage of pALK intensity. Inhibition curves were generated with GraphPad Prism 7.02. Half maximal inhibitory concentrations (IC50s) of different ALK inhibitors are shown in (**B**) as mean ± STD from three independent experiments. * Data cited from [26]. ** Data from [25]. *** Data from [24].

The kinase activity of ALK-Y1278S can be blocked by addition of the ALK TKI crizotinib with an IC50 value around 80 nM (Figure 2A,B), which is consistent with previous work [19,24]. Here, we extended our analysis of ALK-Y1278S employing several different ALK inhibitors, such as lorlatinib, brigatinib, and ceritinib. As readout for ALK activity, we employed phosphorylation of ALK Y1604, which reflects ALK activation [10,25]. We observed that full-length ALK-Y1278S mutant was 8-fold more resistant than wild type ALK to crizotinib (10.0 ± 2.2 nM) as measured by Y1604 phosphorylation [26] (Figure 2A,B). The IC50 of lorlatinib for ALK-Y1278S was 1.7 ± 0.3 nM, approximately 8-fold of that for wild type ALK (0.2 ± 0.03 nM) (Figure 2A,B) [26]. Similarly, the FDA approved second generation ALK TKI ceritinib inhibits the activity of ALK-Y1278S with an IC50 of approximately 40 nM, which is 7.5-fold more resistant than wild type ALK (5.3 ± 0.2 nM) (Figure 2A,B) [25]. The observed IC50 values for brigatinib to abrogate ALK-Y1278S activity is in the single digit nM range, and is only 2-fold higher when compared to wild type ALK, 5.7 nM versus 2.6 nM, respectively [24] (Figure 2A,B). Therefore, the recently developed inhibitors, brigatinib and lorlatinib, display increased efficacy towards ALK-Y1278S when compared to ceritinib and crizotinib.

### 2.3. The Importance of the Hydrogen Bond between Y1278 and C1097

**Figure 3:**
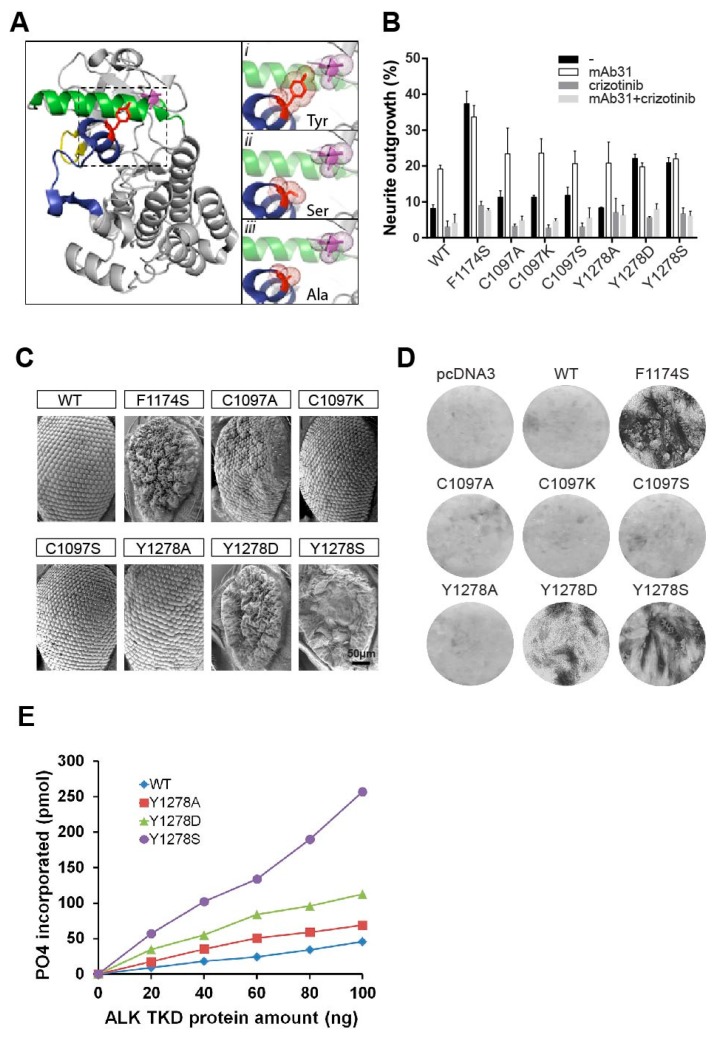
Importance of the Y1278-C1097 hydrogen bond for ALK kinase domain regulation. (**A**, left panel) Structure depicting the ALK kinase domain. αC helix: green; activation loop: dark blue; glycine rich P-loop: yellow. Residues C1097 and Y1278 are shown in purple and red sticks respectively. Image was generated with PyMOL (DeLano Scientific, Palo Alto, CA, USA) using PDB: 3LCT. (Ai, inset) A close up view of the hydrogen bond between Y1278 and C1097. This hydrogen bond is absent when Y1278 is mutated to serine (Aii, inset) or to alanine (Aiii, inset). The selected side chains of residue 1278 (Y/S/A) and C1097 are shown in dotted balls and sticks; (**B**) Neurite outgrowth assay of PC12 cells expressing. Black bars represent untreated cells, white bars denotes cells stimulated with mAb31, dark grey indicate cells treated with 250 nM crizotinib, and light grey denote cells stimulated with mAb31 and treated with crizotinib together; (**C**) Scanning electron microscope images of *Drosophila* eyes ectopically expressing wild type ALK (ALK-WT) and mutant ALK variants (ALK-F1174S, ALK-C1097A, ALK-C1097K, ALK-C1097S, ALK-Y1278A, ALK-Y1278D, and ALK-Y1278S). ALK-F1174S was employed as positive control. Scale bar represents 50 μm; (**D**) Representative focus formation assays for NIH3T3 cells transfected with pcDNA3 empty vector, wild type ALK, or different mutant ALK variants as indicated; (**E**) In vitro kinase assay of the indicated ALK tyrosine kinase domain (TKD) mutants. Substrate phosphorylation activity of wild type ALK-F1174S, ALK-Y1278A, ALK-Y1278D and ALK-Y1278S was assayed employing a peptide mimic of the ALK activation loop. Increasing amounts of ALK TKD proteins were assayed at 30 °C for 30 min.

Since Y1278 is the first tyrosine of the 1278-YRASYY-1283 motif within the activation loop and has been reported to be critical for auto-activation of the ALK kinase domain and transformation ability in the NPM-ALK fusion protein [16,18,27], we wished to investigate the effect of this mutation mechanistically. The crystal structure of inactive ALK reveals a tight interaction between non-phosphorylated Y1278 within the activation loop and the cysteine at position 1097, in the β-turn (Figure 3A). These structural data suggest that phosphorylation of Y1278 upon activation would result not only in the loss of stabilizing hydrogen bond with C1097, but also hypothetically mediate a shift in the αC-helix, thereby facilitating the activation of ALK [13,14]. One hypothesis explaining the ligand independent activation of the ALK-Y1278S neuroblastoma mutation would be that phosphorylation of either Y1278, as in wild type ALK or a mutation, such as Y1278S as in ALK-Y1278S would release the hydrogen bond between residue 1278 and 1097, and thereby initiate kinase activation. To investigate this hypothesis we also mutated residue Y1278 of ALK to Y1278D and to Y1278A. To complement this analysis, we also mutated C1097, in this case replacing the nonpolar cysteine with a basic side chain (lysine), a non-polar side chain (alanine), or an uncharged polar side chain (serine). In agreement with our hypothesis we observed that ALK-Y1278D mimics the ligand independent ALK-Y1278S activating mutation in all of the parameters tested, including neurite outgrowth assays, and the ability to transform NIH3T3 cells (Figure 3B,D). To validate our results from cell-based systems, we also exploited the fruit fly, *Drosophila melanogaster* as a model organism. The different ALK mutants were ectopically expressed in *Drosophila* eye employing the pGMR-Gal4 driver line, which directs protein expression in the eye. This system provides a very clean background [10,28], since the *Drosophila* ALK ligand, Jelly Belly (Jeb), is unable to activate either human or mouse ALK orthologues [10,28,29,30]. In contrast, co-expression with the human ALK ligands (ALKAL1 and 2) results in an activation of the human ALK receptor in the *Drosophila* eye [11]. As expected, ectopic expression of ALK-Y1278S and ALK-Y1278D resulted in a severe rough eye phenotype, similar to the previous reported mutant ALK-F1174S, while ectopic expression of wild type ALK does not produce a rough eye phenotype (Figure 3C). 

In contrast, the mutation of tyrosine 1278 to a non-polar alanine, ALK-Y1278A, resulted in a receptor that was unable to mediate neurite outgrowth, rough eye phenotype or transform NIH3T3 cells (Figure 3B–D). However, upon stimulation with agonist monoclonal antibody the ALK-Y1278A receptor stimulated neurite outgrowth, showing that this mutation did not abrogate ALK kinase activity (Figure 3B).

Moreover, mutational analysis of C1097 to lysine (ALK-C1097K), alanine (ALK-C1097A) or serine (ALK-C1097S) surprisingly failed to generate ligand independent activity as measured by neurite outgrowth, signaling output, rough eye phenotype or NIH3T3 transformation (Figure 3B–D). However, kinase activity was intact, as stimulation of ALK-C1097A, ALK-C1097K, and ALK-C1097S receptors with agonist monoclonal antibody resulted in neurite outgrowth (Figure 3B). These results indicate that mutation of Y1278 to either a charged or polar amino acid, such as serine, leads to constitutive activation of the ALK kinase domain, while substitution of tyrosine to a non-polar alanine on residue 1278, results in an ALK receptor with ligand-dependent character. Similarly, mutations ALK-C1097K, ALK-C1097A, or ALK-C1097S were unable to generate a rough eye phenotype in *Drosophila*. Thus, in an independent model system, we confirm that the mutation of Y1278 to either serine (ALK-Y1278S) or aspartic acid (ALK-Y1278D) led to activation of the ALK kinase domain. This, is in contrast to ALK-Y1278A and various mutations of C1097, which did not appear to be critical for retaining the ALK kinase domain in an inactive form. Thus, phosphorylation of Y1278 may not be necessary for full length ALK activation.

### 2.4. In Vitro Analysis of the ALK-Y1278S/A/D Activation Loop Mutations

To test the importance of the first tyrosine of the 1278-YRASYY-1283 motif—Y1278—we produced the ALK-Y1278S/A/D mutant kinase domains with the baculovirus expression system [16]. These ALK kinase domains (spanning amino acids 1090–1410) contain the C1097 residue, which contacts Y1278 in the crystal structure [13,14]. Purified ALK kinase domains were subsequently subjected to in vitro kinase assays, employing previously described substrate peptide [16]. The kinase domain of wild type ALK and ALK-Y1278A demonstrated weak activity towards the substrate (Figure 3E). In contrast, the ALK-Y1278S kinase domain showed robust activity. The ALK-Y1278D mutant also displayed kinase activity, although not to the same level as ALK-Y1278S. Our kinase assay analyses, which are in agreement with our earlier observations, confirm that the mutation of Y1278 to alanine does not lead to activation of the kinase domain. Taken together, these results also question the importance of the proposed Y1278-C1097 hydrogen bond since Y1278A is unlikely to maintain a hydrogen bond with C1097 (Figure 3A, inset).

### 2.5. Analysis of the Tyrosines in the Activation Loop Motif 1278-YRASYY-1283 of ALK

**Figure 4:**
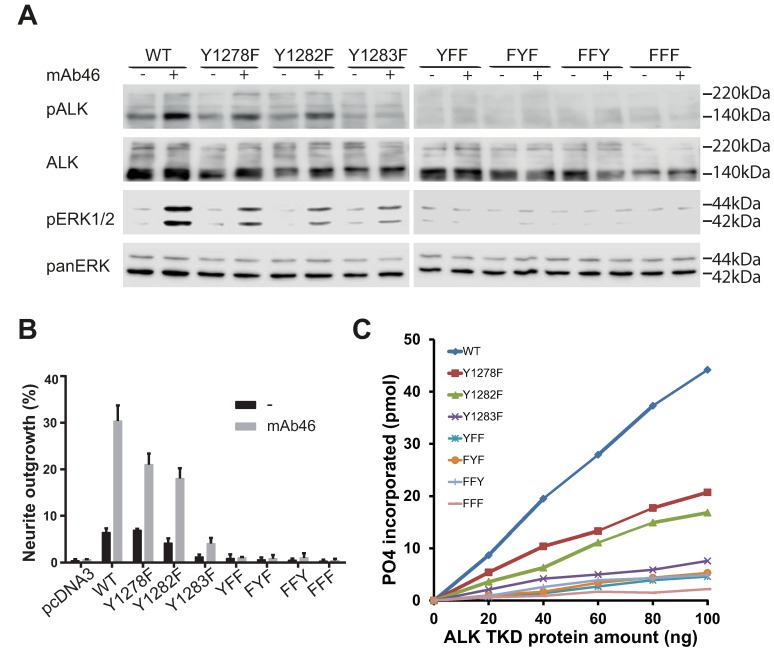
Analysis of the requirement of individual tyrosine residues in the YRASYY activation loop motif for full length ALK activation. (**A**) Detection of phosphorylation of ALK (Y1604) and downstream signalling activity (pERK1/2) of various ALK activation loop mutants by immunoblotting using the indicated antibodies. PanERK was employed as a loading control. Blots are representative of three independent experiments; (**B**) Neurite outgrowth assay of PC12 cells transfected with various ALK mutants, as indicated in the figure, in the presence or absence of mAb46 stimulation. Black bars represent unstimulated cells and grey bars denote cells that are stimulated with mAb46. Bars represent mean percentage ± STD of neurite-carrying cells among GFP-positive cells from three independent experiments; (**C**) In vitro kinase assay of various ALK TKDs. Substrate phosphorylation activity of the ALK TKDs mutated in various combinations of Y1278, Y1282 and Y1283 to phenylalanine was assayed employing a peptide mimic of the ALK activation loop. Increasing amounts of ALK TKD proteins were assayed at 30 °C for 30 min.

The kinase assay results above led to our further investigation of the relative importance of the different tyrosines within the ALK activation loop. A number of mutations were generated within the activation loop of the full length ALK receptor kinase domains (amino acid 1090–1410): ALK-Y1278F, ALK-Y1282F, ALK-Y1283F, as well as the double mutants: ALK-YYY1278,1282,1283YFF, ALK-YYY1278,1282,1283FYF, ALK-YYY1278,1282,1283FFY, and the triple mutant ALK-YYY1278,1282,1283FFF. The various full-length ALK mutants were transfected into PC12 cells, employing wild type ALK as control (Figure 4). 

Upon stimulation with an agonist monoclonal antibody, wild type ALK was phosphorylated on Y1604 and mediated phosphorylation of ERK1/2 (Figure 4A). Mutation of either Y1278 or Y1282 in ALK resulted in a receptor that behaved in a manner similar to wild type in terms of Y1604 phosphorylation and activation of ERK1/2 (Figure 4A). These observations are in agreement with neurite outgrowth assays, where we found that wild type ALK, ALK-Y1278F, and ALK-Y1282F all mediated neurite outgrowth upon stimulation with agonist antibody (Figure 4B). In contrast, mutation of the Y1283 in the 1278-YRASYY-1283 motif failed to promote neurite outgrowth regardless of stimulation (Figure 4B). Moreover, ALK-Y1283F did not display phosphorylation of Y1604, and was unable to activate ERK1/2 (Figure 4A,B). Thus, Y1283 appears to be critical in the context of full length ALK activation and at least two tyrosines, including Y1283, need to be phosphorylated for activity. All of the double and triple mutants in the 1278-YRASYY-1283 motif failed to activate ERK1/2, or to become phosphorylated on Y1604 (Figure 4A). As predicted from these results, they also failed to generate neurite outgrowth regardless of stimulation (Figure 4B). It is clear that these double and triple mutants display greatly reduced activity as compared to either wild type ALK, ALK-Y1278F, and ALK-Y1282F. In conclusion, we find that activation of full length ALK is dependent to a certain extent on the presence of Y1282, but most clearly on Y1283, while Y1278 appears to be less important in the 1278-YRASYY-1283 motif of the activation loop. Further, the mutation of any two of the three tyrosines in the activation loop abrogates the ability of ALK to become activated/phosphorylated or generate neurite outgrowth. 

### 2.6. ALK-Y1283 Is Critical for Wild Type ALK Kinase Activity

The identification of a critical role in full length ALK for Y1283 in the 1278-YRASYY-1283 motif of the activation loop led us to examine this further in vitro employing the corresponding mutant ALK kinase domains purified from baculovirus. We observed that ALK-Y1278F and ALK-Y1282F displayed low levels of activity as compared with the wild type ALK in a kinase assays, however, substantially more than that observed with the ALK-Y1283F mutant kinase domain (Figure 4C). In agreement with the results above, the double mutants: ALK-YYY1278,1282,1283,YFF, ALK-YYY1278,1282,1283FYF, ALK-YYY1278,1282,1283FFY, and the triple mutant ALK-YYY1278,1282,1283FFF displayed negligible levels of kinase activity (Figure 4C). 

**Figure 5:**
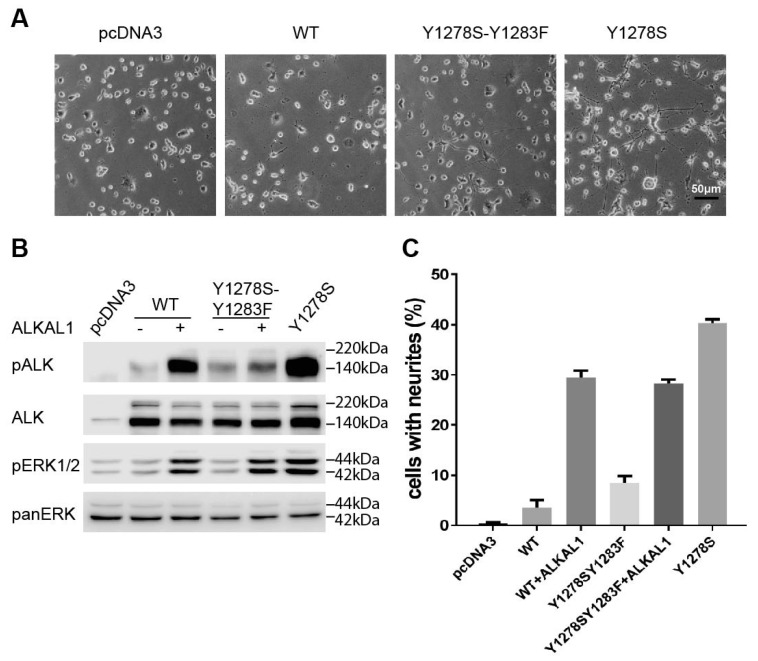
Characterization of the importance of Y1283 for ALK-Y1278S activity. (**A**) Representative images of PC12 cells expressing pcDNA3 empty vector, wild type ALK, ALK-Y1278S, Y1283F or ALK-Y1278S mutants respectively. Scale bar represents 50 μm; (**B**) Detection of phosphorylation of ALK (Y1604) and downstream signalling activity (pERK1/2) of wild type ALK, ALK-Y1278S, Y1283F and ALK-Y1278S in the absence or presence of ALKAL1 ligand by immunoblotting. Blots are representative of three independent experiments; (**C**) Neurite outgrowth assay of PC12 cells transfected with various ALK mutants as indicated in the presence or absence of ALKAL1 stimulation. Bars represent mean percentage ± STD of neurite-carrying cells among GFP-positive cells from three independent experiments.

To further challenge the importance of Y1283 in the activation of ALK a new construct was generated, harbouring the mutations of both Y1278S and Y1283F. Mutation of Y1283F together with Y1278S led to a reduction of neurite outgrowth to levels similar to those observed with unstimulated wild type ALK, and far less than the ALK-Y1278S mutation alone (Figure 1C, Figure 3B and Figure 5A,C). As with wild type ALK, the ALK-Y1278S/Y1283F mutant could be stimulated with the ALK ligand ALKAL1 to generate neurites (Figure 3C). Further, the ALK-Y1278S/Y1283F double mutant failed to stimulate ERK1/2 activation/phosphorylation when compared with the ALK-Y1278S mutation alone. However, upon stimulation with ALKAL1, ALK-Y1278S/Y1283F was able to activated/phosphorylate ERK1/2, although the degree of ALK Y1604 phosphorylation was reduced. Taken together, our data suggests that Y1283 is the most critical tyrosine in the activation loop of ALK for kinase activity, which is in contrast with that reported mechanistically for the NPM-ALK fusion protein [16,18], and our results are more in line with activation mechanisms reported for the InR [15,17].

## 3. Discussion

Here, we describe the experimental analysis of ALK-Y1278S, a mutation in the activation loop of the ALK kinase domain, observed in at least four neuroblastoma patients that harbours both in vitro and in vivo gain of funtion activity, which is in agreement with recently reported observations [19,24,26].

The importance of Y1278 in the mechanism of ALK kinase activation has been investigated in the NPM-ALK fusion protein, where it appears to be phosphorylated first, and then followed by phosphorylation of Y1282 and Y1283 [16,18]. Since ALK fusion proteins are activated by dimerization via their fusion partners, they lack the regulation imposed by the extracellular, transmembrane, and juxtramembrane domains of the ALK RTK, it has been unclear whether the mechanism described for NPM-ALK also applies for full length ALK. Predictions from the ALK crystal structure [13,14] infer that Y1278 makes a critical connection with C1097 in the β-turn, stabilizing the inactive form of the kinase. This explanation fits well with the appearance of Y1278S as an activating mutation in neuroblastoma. However, the analysis presented here suggested that this interaction is not critical, since the mutation of C1097 to either alanine, lysine, or serine does not result in ligand independent kinase activation. Importantly, these ALK-C1097A, ALK-C1097K, and ALK-C1097S mutant receptors can still be activated by simulation through the extracellular domain. Thus, the hydrogen bond between Y1278 and C1097 appears to be less important in the regulation of ALK kinase activation than previously predicted. The structural studies published so far provide limited information regarding the mechanism of ALK activation, and the structure of the ALK-Y1278S mutant kinase domain would contribute important information to our understanding.

In addition to examining the importance of the Y1278:C1097 interaction, we have analysed the importance of Y1278 relative to the other tyrosines in the 1278-YRASYY-1283 motif of the activation loop. Our data suggests that phosphorylaton of Y1278 is not crucial for the autoactivation of the full length ALK receptor upon stimulation since an ALK-Y1278A mutant receptor can still be activated. This is in contrast to the findings of Pina and Gambacorti-Passerinei, who were studying the activation of the NPM-ALK fusion protein [16,18]. In the NPM-ALK fusion protein, they highlighted the importance of phosphorylation of the first tyrosine, which they reported as necessary for the transformation activity of NPM-ALK, as well as for the NPM-ALK STAT3 interaction [18]. Our investigations suggest that Y1283, rather than Y1278, is necessary for the phosporylation/activation/enzymatic activity of the wild type ALK kinase, downstream signling to ERK1/2 and in cellular neurite outgrowth assay. However, Y1283 does not seem to be critical in the context of the ALK-Y1278S mutant, since the ALK-Y1278S /Y1283F double mutant no longer displays constitutive activation but is activated upon stimulation with ALKAL1 ligand. Thus an activating mutation of Y1278 is able to partially compensate for lack of Y1283 in the presence of a stimulating ligand. 

## 4. Material and Methods

### 4.1. Antibodies and Inhibitors

The primary antibodies used were: anti-pan-ERK from BD Transduction Laboratories (Franklin Lakes, NJ, USA), anti-pALK (Y1604), anti-pAKT (S473), anti-AKT, anti-ERK1/2 and anti-pERK1/2 (T202/Y204) from Cell Signalling Technology (Danvers, MA, USA). The monoclonal antibodies mAb31, mAb46 and mAb135 have been described previously [28,31]. The horseradish peroxidase conjugated secondary antibodies goat anti-rabbit IgG and goat anti-mouse IgG were purchased from Thermoscientific (Waltham, MA, USA). The ALK inhibitor crizotinib was purchased from ChemExpress (Shanghai, China). Other ALK inhibitors were purchased from Selleck Chemicals (Houston, TX, USA). Human ALK ligand ALKAL1 was produced by GenScript (Piscataway, NJ, USA).

### 4.2. Generation of ALK Mutant Constructs

Construction of wild type ALK, ALK-F1174L, and ALK-F1174S plasmids has been described earlier [28]. ALK mutants employed in this study namely C1097A/S/K and Y1278S/A/D were created by Eurofins MWG/operon (Ebersberg, Germany). Mutations in the YRASYY motif of the activation loop: Y1278F, Y1282F, Y1283F, double mutants YFF, FYY, FYF, and triple mutant FFF were generated using QuikChange site-directed mutagenesis kit from Agilent (Santa Clara, CA, USA). ALK mutant Y1278S-Y1283F was generated based on ALK-Y1278S using QuikChange site-directed mutagenesis kit. Manufacturer’s protocol was followed. The mutations generated above were all confirmed by sequencing from both directions.

### 4.3. Neurite Outgrowth Assay

PC12 cells (2 × 10^6^) were co-transfected with 0.5 µg of pcDNA3, ALK, or ALK variants as indicated, and 0.5 µg of pEGFP-N1 by electroporation using Amaxa electroporator (Amaxa Biosystems, Cologne, Germany). Cells were resuspended in 100 µL of Ingenio electroporation solution (Mirus Bio LCC, Madison, WI, USA) prior to transfection. After transfection, cells were kept in MEM supplemented with 7% horse serum and 3% fetal bovine serum. The transfected cells were then seeded into 24 well plates with or without 1 µg/mL of the activating monoclonal antibody mAb31 or mAb46 or ALK ligand ALKAL1, as indicated in the figures. In some cases, crizotinib (250 nM final concentration) was employed. After 48 h of incubation, the fraction of GFP positive and neurite carrying cells versus GFP positive cells was observed under a Zeiss Axiovert 40 CFL microscope (Thornwood, NY, USA). To be judged as a neurite carrying cell, the neurite of the cell should be at least twice the diameter of a normal cell body. Experiments were performed in triplicates, and each sample within an experiment was performed in triplicates.

### 4.4. Cell Lysis and Immunoblotting

PC12 cells expressing wild type ALK or ALK variants were serum starved for 36 h prior to stimulation with 1 µg/mL of mAb31 or mAb46 or ALKAL1 ligand as indicated for 30 min [30,31,32]. Empty vector transfected cells were used as negative control. Cells were then washed with ice-cold 1× PBS prior to harvest in lysis buffer (25 mmol/L of Tris-Cl, pH7.5, 150 mmol/L of NaCl, 1% (*v*/*v*) Triton X-100, 1 mmol/L of DTT, protease inhibitor cocktail tablet). Cell lysates were cleared by centrifugation at 14,000 rpm for 15 min at 4 °C. Samples were boiled in SDS sample buffer and were analyzed by immunoblotting with the indicated antibodies. ALK downstream activation was detected by anti-pERK1/2 antibody and/or anti-pAKT (S473) antibody. Pan-ERK or ERK1/2 was used as loading control. ALK phosphorylation was checked by anti-pALK (Y1604) antibody. Total ALK was detected with ALK mAb135.

### 4.5. Transformation Assay

NIH3T3 cells (4.5 × 10^4^) were seeded in collagen-coated 12-well plates a day before the transfection. The cells were transfected for 6 h with 1.75 µg pcDNA3-hALK and 4.4 µL of Lipofectamine 2000 (Invitrogen, Paisley, UK) in 0.3 mL of opti-MEM. A day later, 3/5th of cells were transferred to 6-well plates and were maintained in DMEM with 10% heat inactivated fetal bovine serum and 0.5 mg/mL of G418 until the cells reached confluence. After which the cells were kept in DMEM with 5% heat inactivated Fetal bovine serum and 0.25 mg/mL of G418 for about 10 days. The cells were then fixed using methanol and stained with 0.25% crystal violet. The plates were dried and the focus density was photographed and quantified.

### 4.6. ALK Inhibitor Profiles on ALK-Y1278S Mutant 

PC12 cells (2 × 10^6^) were transfected by electroporation in an Amaxa electroporator using 1.5 μg of ALK-Y1278S plasmids and 100 μL of Ingenio electroporation solution (Mirrus Bio LCC, Madison, WI, USA). After transfection, cells were transferred to DMEM supplemented with 7% horse serum and 3% FBS. Cells from 4 same electroporations were pooled together, well mixed, and were equally seeded into 11 wells of a 24-well plate. 48 h after transfection, cells were treated with serial dilutions of crizotinib, lorlatinib, ceritinib or brigatinib respectively (all from 0 to 1000 nM) for 2 h. Cells were then washed with cold 1× PBS and lysed with 1× SDS sample buffer and samples were boiled at 95 °C for 5 min. Phospho-ALK (Y1604) antibody was used to detect ALK phosphorylation and ALK mAb135 was used to detect total ALK. The intensity of pALK (Y1604) and total ALK bands was quantified with Image Studio Lite 3.1 software. Data were normalized to the 0nM inhibitor samples as percentages of pALK intensity. IC50 values were calculated by fitting data to a log (inhibitor) vs. normalized response (variable slope) equation in GraphPad Prism 7.02. All data are shown as means ± STD from three independent experiments.

### 4.7. Generation of Recombinant ALK TKD Proteins 

DNA encoding ALK residues 1090–1416, which includes whole tyrosine kinase domain (TKD), was amplified with Phusion High-Fidelity DNA polymerase (ThermoScientific, Waltham, MA, USA) and cloned into pFastBac/NT-TOPO vector (Invitrogen, Carlsbad, CA, USA) for expression of 6x histidine-tagged recombinant ALK TKD proteins in Sf21 cells. Recombinant baculovirus was generated using the Bac-to-Bac baculovirus expression system (Invitrogen, Carlsbad, CA, USA). Sf21 cells were infected with P2 virus stock for three days at 27 °C, and then lysed in native Ni-NTA lysis buffer (50 mM NaH_2_PO_4_ pH 8.0, 300 mM NaCl, 10 mM imidazole, 1% Triton X-100, and protease inhibitor cocktail). His-tagged protein was recovered from the lysis supernatant using Ni-NTA Superflow beads (Qiagen, Hilden, Germany). After 2× wash, the resins were incubated in 50 mM Tris-Cl pH 7.4, 150 mM NaCl, 10 mM MgCl_2_ and 2 mM ATP at 30 °C for 1 h to achieve further ALK TKD autophosphorylation. After 2× additional washes, ALK TKD proteins were eluted and their concentrations were determined by absorbance at 280 nm. Purity of ALK TKD proteins was assessed by SDS-PAGE and Coomassie blue staining.

### 4.8. In Vitro Kinase Assays 

Analysis of substrate phosphorylation by ALK TKD employed a peptide mimic of the ALK activation loop with sequence: ARDIYRASYYRKGGCAMLPVK (Caslo, Danmark), referred to as YYY peptide [16]. Assays were performed using radiolabeled ATP, as described previously [33]. Different amount of ALK TKD proteins were used with fixed concentration of ATP and peptide at 0.1 mM and 0.2 mM, respectively. Assays were conducted at 30 °C for 30 min and the incorporation of ^32^P from γ-^32^P ATP into the substrate was detected with Wallac 1414 Liquid Scintillation Counter (Perkin Elmer, Waltham, MA, USA). 

### 4.9. Expression of Human ALK Mutants in Drosophila Eye 

The Gal4-UAS expression system was used for the ectopic expression of human ALK in *Drosophila* eye [34]. cDNA encoding wild type ALK and mutant ALK were subcloned with EcoRI and NotI from pcDNA3-ALK and the resulting fragments were ligated into the EcoRI-NotI site of the pUAST vector. Transgenic flies carrying pUAST-ALK were generated by BestGene, Inc. (Chino Hills, CA, USA), and crossed with *pGMR-Gal4*, an eye specific driver fly line. 

### 4.10. Expression of Human ALK Mutants in Drosophila Eye 

The Gal4-UAS expression system was used for the ectopic expression of human ALK in *Drosophila* eye [34]. cDNA encoding wild type ALK and mutant ALK were subcloned with EcoRI and NotI from pcDNA3-ALK and the resulting fragments were ligated into the EcoRI-NotI site of the pUAST vector. Transgenic flies carrying pUAST-ALK were generated by BestGene, Inc. (Chino Hills, CA, USA), and were crossed with *pGMR-Gal4*, an eye specific driver fly line. 

## 5. Conclusions

In conclusion, we observe that the mutation of Y1278 to serine or aspartic acid results in kinase domain activation, however the proposed Y1278:C1097 interaction does not appear to play a critical role. In terms of the activation loop mechanism, we find that while phosphorylation of Y1278 is sufficient for ALK kinase activation, it is not a critical requirement; the third tyrosine (Y1283) in the 1278-YRASYY-1283 motif of the activation loop appears to be critical for wild type ALK kinase activity. Our findings here, studying the full length ALK receptor, as well purified ALK kinase domains, support a mechanism of activation that differs from the NPM-ALK fusion protein and is more in line with those observed for the InR, where the second and third tyrosines (Y1162 and Y1163) are phosphorylated prior to the first (Y1158) [15,17,35].
